# Case Report: A rare case of myopic macular pit mimicking duplication of the optic disc

**DOI:** 10.3389/fmed.2026.1714514

**Published:** 2026-01-22

**Authors:** Sirui Zhou, Mingjue Hu, Yi Sun, Zhanfeng Wang, Ting Luo

**Affiliations:** Department of Ophthalmology, The Third People’s Hospital of Chengdu, Chengdu, Sichuan, China

**Keywords:** case report, duplication of the optic disc, multimodal imaging, myopic macular pit, pathologic myopia, scleral pit

## Abstract

**Introduction:**

Myopic macular pit (MMP) is a rare manifestation of pathologic myopia. Here, we report a case of MMP with optic disc-like features that posed a diagnostic challenge and was misinterpreted as duplication of the optic disc.

**Case presentation:**

A 69-year-old man with high myopia presented with visual impairment. Examination of his right eye revealed an axial length of 30.27 mm and best-corrected visual acuity of counting fingers at 10 cm. Fundus examination revealed a disc-sized pit within the macular atrophy, accompanied by radially oriented retinal and choroidal vessels. Owing to its disc-like appearance, the lesion was initially misdiagnosed as a duplication of the optic disc. Subsequent multimodal imaging established the correct diagnosis of MMP: orbital magnetic resonance imaging demonstrated a single optic nerve, fundus fluorescein angiography and indocyanine green angiography showed vascular filling consistent with short posterior ciliary arteries, and optical coherence tomography revealed chorioretinal atrophy and sharply curved scleral excavation.

**Conclusion:**

MMP should be differentiated from true and pseudoduplication of the optic disc. Multimodal imaging plays a pivotal role in resolving this diagnostic challenge.

## Introduction

Myopic macular pit (MMP), also known as myopic scleral pit (1), is a rare manifestation of pathologic myopia (2). First described by Ohno-Matsui et al. (3), few cases have been reported worldwide. MMP typically occurs in highly myopic eyes with an axial length ≥30 mm, most often in individuals over 50 years of age, and may be unilateral or bilateral. Its pathogenesis involves a focal scleral expansion (4). All reported lesions have been located within an area of severe macular chorioretinal atrophy, frequently accompanied by posterior staphyloma. MMP can also be associated with other complications of pathologic myopia, such as retinoschisis, epiretinal membranes, macular holes, retinal lattice degeneration, lacquer cracks, and choroidal neovascularization (1–3, 5, 6). Clinically, patients frequently present with severe visual impairment, with approximately half of the affected eyes exhibiting a best-corrected visual acuity (BCVA) worse than 1.0 logMAR (1, 2, 4–7). On fundoscopic examination, MMP appears as one or more pit-like lesions within macular chorioretinal atrophy, always inferior or temporal to the macula, often close to the inferior vascular arcade (4). In certain cases, the retinal or choroidal vessels traverse the pit, creating an optic disc-like appearance. This led Ahn et al. (7) to describe the phenomenon as an “acquired pseudoduplication of the optic disc”. Consequently, MMP can be misdiagnosed as a duplication of the optic disc, especially when its morphology is disc-like and assessment relies solely on ophthalmoscopy.

Here, we present a rare case in which the disc-like appearance of MMP initially suggested a misdiagnosis of duplication of the optic disc. Multimodal imaging, including magnetic resonance imaging (MRI), fundus fluorescein angiography (FFA), indocyanine green angiography (ICGA), and optical coherence tomography (OCT), ultimately confirmed the correct diagnosis of MMP.

## Case presentation

**Figure 1:**
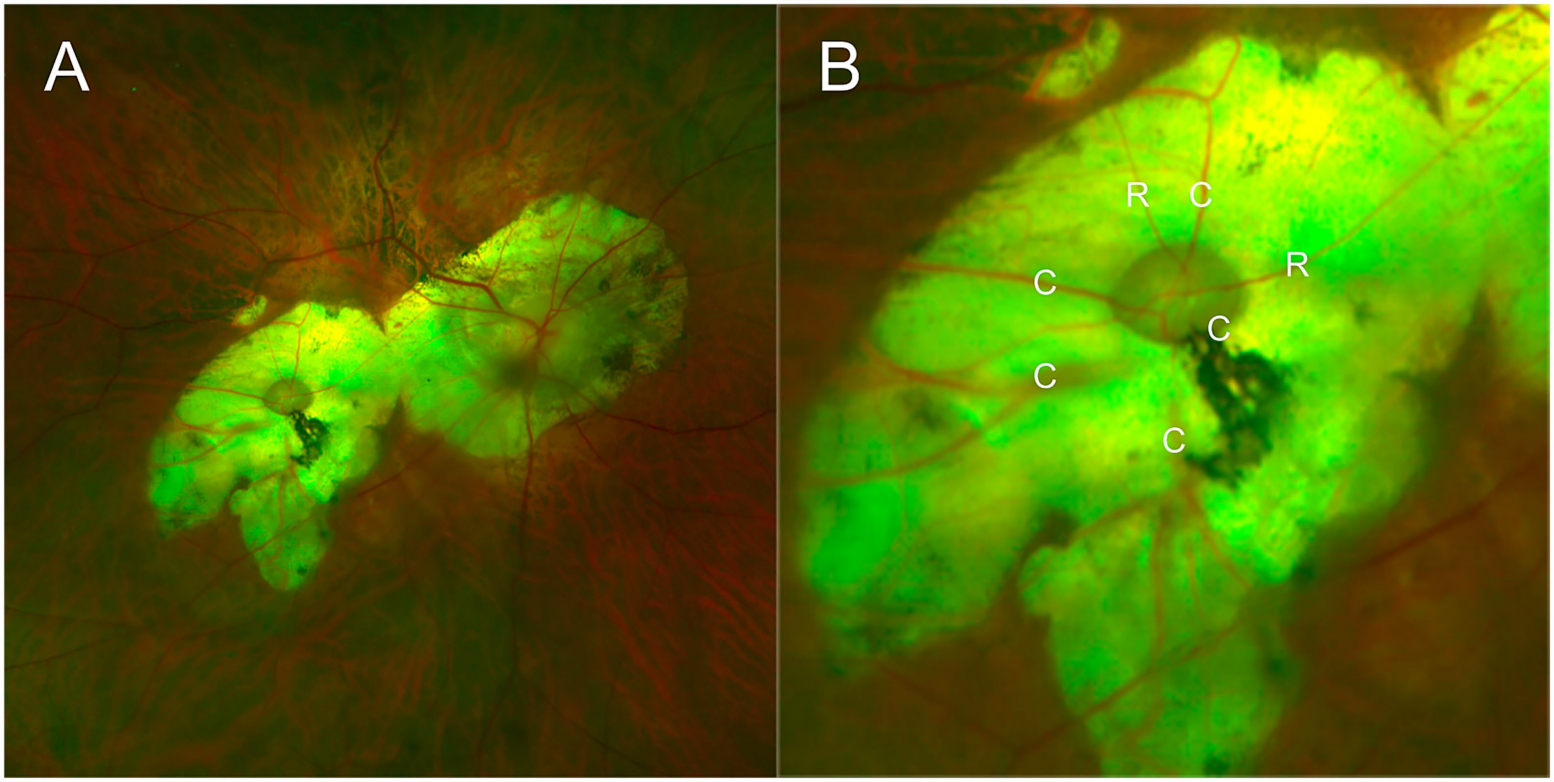
Fundus appearance of the disc-like lesion in the right eye. **(A)** A well-defined, grayish-brown pit, approximately one disc diameter in size, is located within the macular atrophic zone, from which multiple retinal and choroidal vessels radiate. **(B)** A detailed view shows the vascular configuration around the pit. The letter R in the image indicates secondary branches of the superotemporal retinal vessel that cross the base of the pit. The letter C represents multiple choroidal vessels radiating from the pit to the periphery.

A 69-year-old man with a history of high myopia presented with progressive loss of vision. His medical history included diabetes mellitus, with no other significant ocular or systemic conditions. On examination, BCVA was counting fingers at 10 cm in both eyes. Axial length measurements (IOL Master 700; Carl Zeiss Meditec AG, Jena, Germany) were 30.27 mm in the right eye and 31.00 mm in the left. Intraocular pressure (Reichert 7; Reichert Inc., New York, United States) was 15 mmHg in the right eye and 13 mmHg in the left. Mild bilateral lens opacities were noted. Fundus examination revealed extensive chorioretinal atrophy involving the peripapillary and macular regions bilaterally, with inferior displacement of the macular zone in the right eye. Within the atrophic macula, a well-defined, grayish-brown pit measuring approximately 1 disc diameter (DD) was identified (Figure 1A). Several vessels were distributed around the pit. A secondary branch from the superotemporal retinal artery was observed entering the nasal margin, crossing the base, and exiting the temporal margin, while multiple choroidal vessels emerged from the pit and then crossed the atrophic region (Figure 1B). Given this disc-like morphology, the lesion was initially suspected to be a duplication of the optic disc.

**Figure 2:**
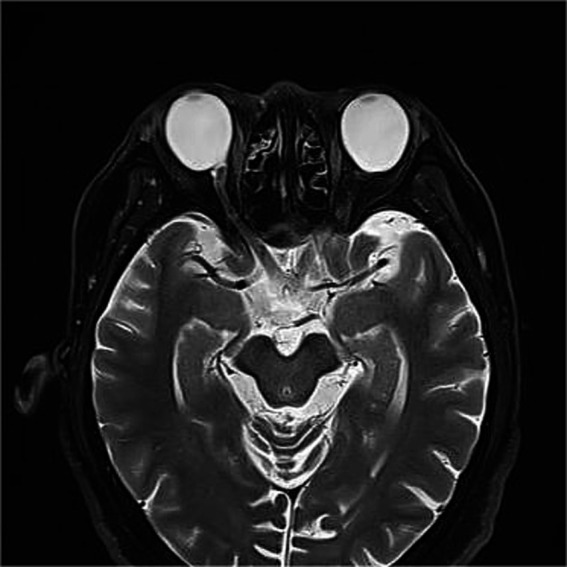
Orbital magnetic resonance imaging (MRI) findings. Orbital MRI demonstrates axial elongation with posterior staphyloma in both eyes. Only a single optic nerve is identified posterior to each globe.

**Figure 3:**
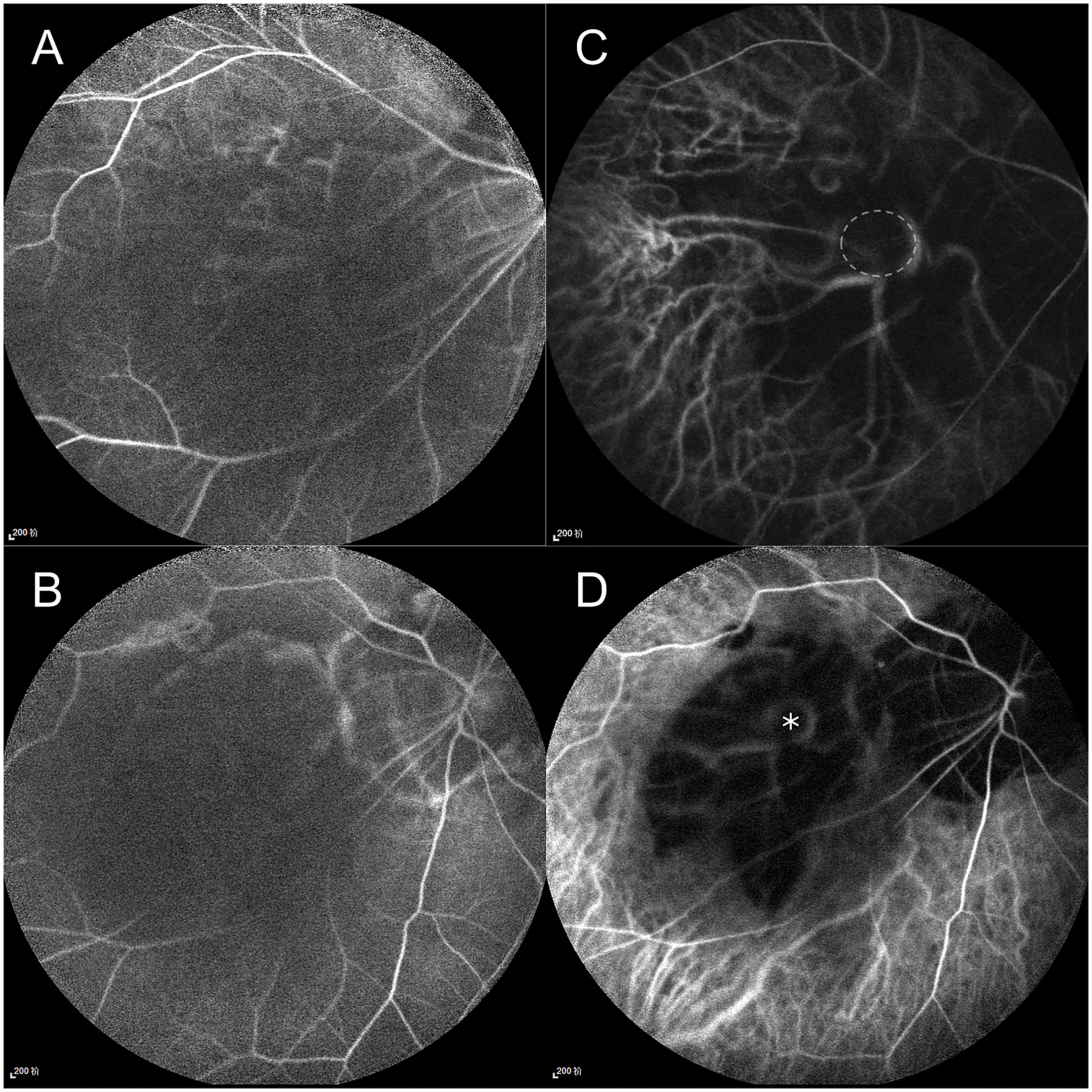
Fundus fluorescein angiography (FFA) and indocyanine green angiography (ICGA) findings. FFA: **(A)** At 24 s, retinal vessels are visible at the optic disc but absent within the pit. **(B)** At 3 min 35 s, the optic disc shows hyperfluorescence, contrasting with the non-hyperfluorescent pit. ICGA: **(C)** At 20 s, short posterior ciliary arteries (SPCAs) originate from the pit. The margin of the pit is outlined by a white circle. **(D)** At 3 min 35 s, ring-shaped staining is observed along the pit margin; the pit location is marked with an asterisk.

**Figure 4:**
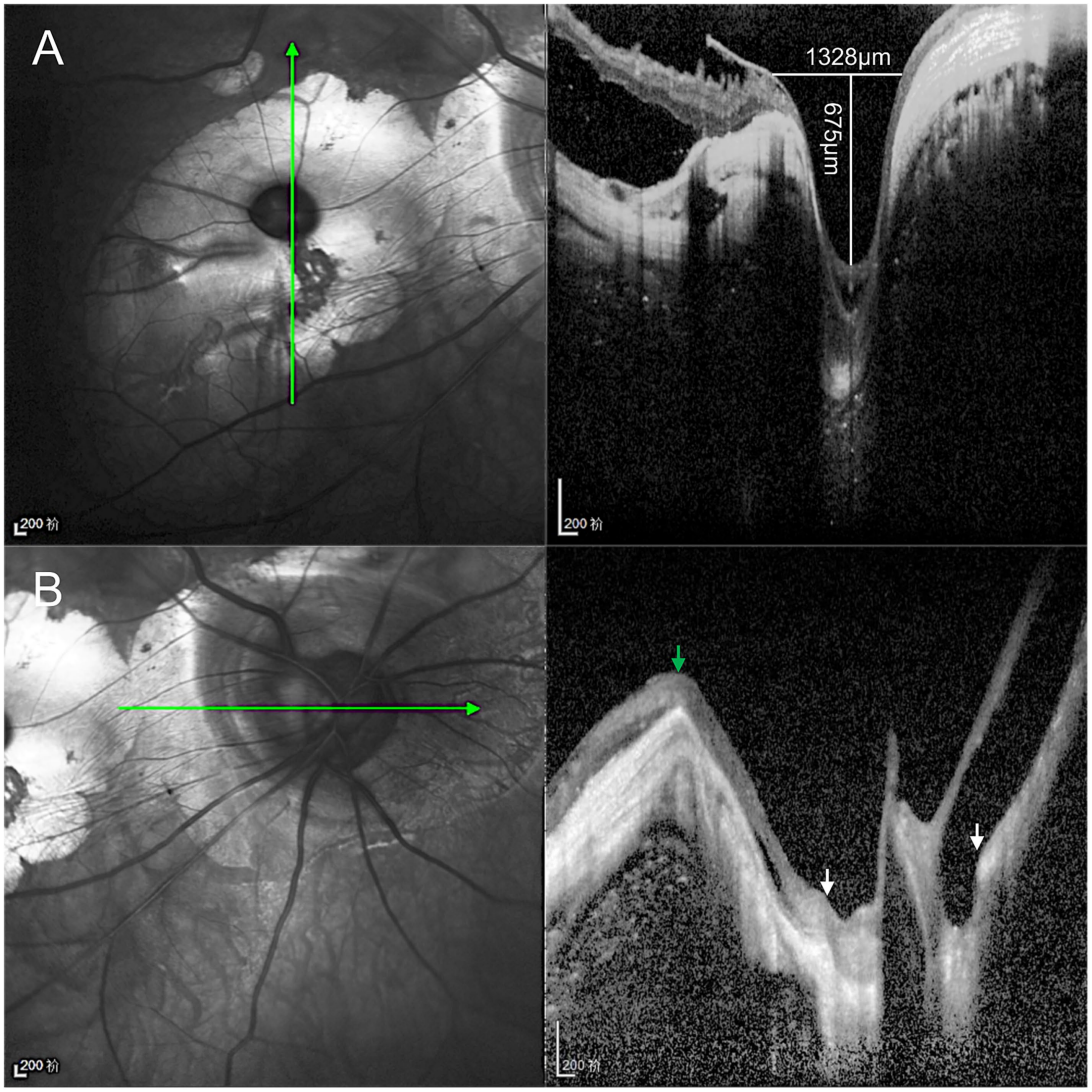
Optical coherence tomography (OCT) characteristics of the disc-like pit and true optic disc. **(A)** The disc-like pit is located within the posterior staphyloma, where the scleral curvature changes sharply, forming a focal deep excavation measuring 1,328 μm in width and 675 μm in depth. The sclera within the pit is thinned, the choroid is absent, and retinoschisis is observed at both the base and the margin of the excavation. **(B)** The true optic disc is located at the bottom of the peripapillary staphyloma (between white arrows), accompanied by a temporal scleral ridge (green arrow) and nasal retinoschisis, and its excavation is shallower and flatter than that of the disc-like lesion.

Given the extreme rarity of duplication of the optic disc, multimodal imaging, including MRI, FFA, ICGA, and OCT, was performed for further characterization. Orbital MRI revealed marked axial elongation with posterior staphyloma in both eyes; however, only a single optic nerve was identified (Figure 2). On FFA (HRA Spectralis; Heidelberg Engineering, Heidelberg, Germany), retinal vessels were clearly visible in the optic disc at 24 s, while the pit showed no obvious vessels (Figure 3A). At 3 min 35 s, the optic disc exhibited hyperfluorescence, in contrast to the non-hyperfluorescent pit (Figure 3B). ICGA (HRA Spectralis, Heidelberg Engineering, Heidelberg, Germany) demonstrated that, at 16 s, the vessels around the pit filled synchronously with other choroidal vessels, a pattern consistent with perfusion by the short posterior ciliary arteries (SPCAs). At 20 s, the choroidal vessels were clearly visualized, followed by the initial filling of the retinal arteries (Figure 3C). At 3 min 35 s, ring-shaped staining was observed along the pit margin (Figure 3D). OCT (Spectralis OCT; Heidelberg Engineering, Heidelberg, Germany) revealed a localized, deep excavation within the area of posterior staphyloma and chorioretinal atrophy, characterized by an abrupt change in the scleral curvature. The pit measured 1,328 μm in width and 675 μm in depth (Figure 4A). Within the pit, the sclera appeared thin, the choroid was absent, and the retina demonstrated schisis at its base and margin. In contrast, the true optic disc, located in the peripapillary staphyloma of the same eye, was associated with a temporal scleral ridge and nasal retinoschisis; its excavation was shallower and flatter than that of the disc-like pit (Figure 4B). Based on these multimodal imaging findings, the disc-like lesion was definitively diagnosed as MMP. The patient subsequently underwent bilateral cataract surgery to address lens opacity. No intervention was performed for the fundus lesion. Postoperatively, the BCVA improved to 1.70 logMAR and remained stable along with the OCT findings, at the one-year follow-up.

## Discussion

We report a rare manifestation of pathologic myopia that, while morphologically consistent with previous reports, presents the most striking resemblance to a true optic disc to date. Its defining features include a pit comparable in size to the optic disc, an adjacent atrophic zone mimicking peripapillary atrophy, and a central vascular pattern similar to that of the optic disc. However, careful comparison revealed subtle but important fundoscopic distinctions. The MMP had a horizontally oval shape and lacked a neuroretinal rim. More critically, multimodal imaging provided further evidence for this distinction. OCT revealed hyperreflective scleral tissue at the base of the MMP, which was continuous with the surrounding sclera. In contrast, the true optic disc exhibited a normal lamina cribrosa structure (8, 9). Moreover, the MMP excavation was deeper and steeper than that of the optic disc. FFA and ICGA confirmed that the vessels emerging from the pit originated from the SPCAs, rather than the central retinal artery. Furthermore, the pit lacked the characteristic hyperfluorescence observed in the true optic disc on FFA. These distinctions were not appreciable using ophthalmoscopy alone. Without multimodal imaging, such lesions risk being misdiagnosed as duplication of the optic disc. This case highlights the importance of multimodal imaging in establishing a correct diagnosis.

Duplication of the optic disc, a rare congenital anomaly of optic nerve development, is classified as either a true duplication or pseudoduplication (10). Diagnostic criteria for true duplication include imaging evidence of the two optic nerves and two optic foramina, fluorescein angiography demonstration of an independent vascular supply, and visual field confirmation of nerve fiber distribution from the accessory disc (11, 12). Although sporadic reports have been supported by autopsy, radiography, and ultrasonography (11, 13–15), definitive *in vivo* confirmation by CT or MRI remains underinvestigated. In our case, MRI revealed no second optic nerve, thereby excluding true duplication and suggesting pseudoduplication. To our knowledge, this is the first study to employ MRI to distinguish MMP from these entities.

Pseudoduplication of the optic disc is typically attributed to congenital chorioretinal coloboma resulting from incomplete closure of the embryonic optic fissure (10, 16). As the optic fissure is located on the ventral surface of the developing optic vesicle (17), the resulting pseudodisc is typically inferior to the true disc (16). It may have an independent retinal vasculature or share vessels with the true optic disc, often interconnected by anastomoses (10, 18–22). While MMP may mimic pseudoduplication, both are fundamentally distinct. In terms of etiology and pathogenesis, MMP is an acquired lesion secondary to pathologic myopia, associated with short posterior ciliary arteries and lacking retinal anastomoses. The disc-like lesion in our case was located within the macular region and was nearly horizontal to the true optic disc. On OCT, MMP exhibited consistent characteristics, typically presenting as a deep scleral excavation, measuring less than 1.0 DD, with scleral dehiscence in some cases (1, 4, 23, 24). The outer retina and choroid were absent, while the inner retina was thinned, lost, or showed overlying schisis (4). In contrast, the pseudoduplications exhibited high morphological variability. Their size ranged from 0.5 to 3.0 DD (7), and their appearance spanned a spectrum from MMP-like presentations to small colobomatous defects, huge crater-like depressions, or ectatic coloboma (10, 18, 19, 21, 25–28).

Consistent with previous reports, the MMP in this case was located within an area of chorioretinal atrophy, precisely at the scleral entry site of the distal SPCAs (2, 5–7, 29, 30). This process may share a similar underlying mechanism with conus pit formation in pathologic myopia (31). The biomechanical strength of the sclera within the atrophic area is diminished, predisposing it to expansion at the vessel canals (2, 5–7). Rather than established MMP, Xie et al. (32) found slight sclera bowing around the vascular entry points, suggesting a possible early change. Furthermore, the uneven protrusion of the staphyloma displaced the macular region inferiorly, while countertraction from the sclera and vitreous, along with the rigidity of the inner limiting membrane, potentially contributed to the development of retinoschisis adjacent to the pit (33). However, it remains unclear why MMP was always observed inferior or temporal to the macula (4).

In most cases, no intervention is required. Although patients with MMP frequently exhibit poor visual acuity, this is largely due to chorioretinal atrophy and associated complications rather than MMP itself. This observation is supported by Zhang et al. (1), who found no correlation between BCVA and the size of the macular pits. However, sight-threatening complications, such as retinal detachment, choroidal neovascularization, macular holes, or epiretinal membranes, warrant prompt and specific treatment.

## Conclusion

In conclusion, certain cases of MMP, particularly those within areas of chorioretinal atrophy and associated with disc-like vascular patterns, may closely mimic duplication of the optic disc, posing a potential diagnostic challenge. Clinicians should maintain a high index of suspicion for this entity and employ multimodal imaging to accurately differentiate MMP from duplication of the optic disc and other mimicking conditions, thereby avoiding misdiagnosis. Given the inherent limitations of a single case, larger case series with long-term follow-up are warranted to further elucidate the pathogenesis, clinical course, and relationship of MMP to other complications of pathologic myopia.

## Data Availability

The raw data supporting the conclusions of this article will be made available by the authors, without undue reservation.

## References

[1] Zhang W Zhang Y Xu J Dan H Li X Song Z. A physical sign of pathological myopia: myopic scleral pit. BMC Ophthalmol. (2023) 23:114. doi: 10.1186/s12886-023-02847-y, 36949450 PMC10031946

[2] Fogel Levin M Freund KB Gunnemann F Greaves G Sadda S Sarraf D. Myopic macular pits: a case series with multimodal imaging. Can J Ophthalmol. (2023) 58:125–30. doi: 10.1016/j.jcjo.2021.09.003, 34626545

[3] Ohno-Matsui K Akiba M Moriyama M Ishibashi T Hirakata A Tokoro T. Intrachoroidal cavitation in macular area of eyes with pathologic myopia. Am J Ophthalmol. (2012) 154:382–93. doi: 10.1016/j.ajo.2012.02.010, 22541655

[4] Pedinielli A Souied EH Perrenoud F Leveziel N Caillaux V Querques G. *In vivo* visualization of perforating vessels and focal scleral ectasia in pathological myopia. Invest Ophthalmol Vis Sci. (2013) 54:7637–43. doi: 10.1167/iovs.13-12981, 24194186

[5] Ohno-Matsui K Akiba M Moriyama M. Macular pits and scleral dehiscence in highly myopic eyes with macular chorioretinal atrophy. Retin Cases Brief Rep. (2013) 7:334–7. doi: 10.1097/ICB.0b013e318290d6f5, 25383824

[6] Vadivelu JP Shah A Khetan V Lingam G. Multimodal imaging to differentiate myopic macular pit and localized deep staphyloma in high myopia. Indian J Ophthalmol. (2019) 67:1173–4. doi: 10.4103/ijo.IJO_1577_18, 31238444 PMC6611306

[7] Ahn SJ Woo SJ Hwang JM. Acquired pseudoduplication of the optic disc in pathologic myopia. Optom Vis Sci. (2014) 91:e177–84. doi: 10.1097/OPX.0000000000000300, 24927139

[8] Sanfilippo PG Cardini A Hewitt AW Crowston JG Mackey DA. Optic disc morphology—rethinking shape. Prog Retin Eye Res. (2009) 28:227–48. doi: 10.1016/j.preteyeres.2009.05.004, 19520180

[9] Zhang X Jiang J Kong K Li F Chen S Wang P . Optic neuropathy in high myopia: glaucoma or high myopia or both? Prog Retin Eye Res. (2024) 99:101246. doi: 10.1016/j.preteyeres.2024.101246 38262557

[10] Chen S Liu X Zhong J. Uveal colobomas with pseudo-duplication of the optic disc in both eyes: imaging by ultra-widefield swept-source optical coherence tomography angiography: a case report. J Med Case Rep. (2024) 18:366. doi: 10.1186/s13256-024-04676-z, 39129019 PMC11318245

[11] Lamba PA. Doubling of the papilla. Acta Ophthalmol. (1969) 47:4–9. doi: 10.1111/j.1755-3768.1969.tb05604.x, 4979346

[12] Brink JK Larsen FE. Pseudodoubling of the optic disc. A fluorescein angiographic study of a case with coloboma. Acta Ophthalmol. (1977) 55:862–70. doi: 10.1111/j.1755-3768.1977.tb08285.x, 578644

[13] Narendiran DPP Kamaraj DP Agarwal DA. True optic disc doubling with associated optic nerve doubling. Am J Ophthalmol. (2021) 230:e2. doi: 10.1016/j.ajo.2021.06.024, 34283977

[14] Chen N Balas M Arjmand P. True duplication of the optic nerve. Ophthalmology. (2025) 132:e102. doi: 10.1016/j.ophtha.2024.08.031, 39352327

[15] Ren Y Xiao T. Doubling of optic disc. Br J Ophthalmol. (2008) 92:1151–2. doi: 10.1136/bjo.2007.125559, 18653611

[16] Jeng-Miller KW Cestari DM Gaier ED. Congenital anomalies of the optic disc: insights from optical coherence tomography imaging. Curr Opin Ophthalmol. (2017) 28:579–86. doi: 10.1097/ICU.0000000000000425, 28817389

[17] Patel A Sowden JC. Genes and pathways in optic fissure closure. Semin Cell Dev Biol. (2019) 91:55–65. doi: 10.1016/j.semcdb.2017.10.010, 29198497

[18] Pereira A Orr S Choudhry N. Pseudoduplication of the optic disc. Can J Ophthalmol. (2023) 58:e115. doi: 10.1016/j.jcjo.2022.08.007, 36108791

[19] Xu YX Fu YH Wen M Lu F Yang Y Sha J . A case report of bilateral pseudo-doubling of the optic discs. Ann Palliat Med. (2022) 11:2510–5. doi: 10.21037/apm-21-1087, 34498472

[20] Fazel F Zarrin Y Fazel M. Optic disc pseudoduplication accompanied with macular hole. Indian J Ophthalmol. (2020) 68:2530. doi: 10.4103/ijo.IJO_927_20, 33120675 PMC7774138

[21] Huang L Zhang Q Jin H Zhao P. Pseudoduplication of the optic disc initially resembling a bifurcated optic nerve in a strabismus child: a case report. BMC Ophthalmol. (2020) 20:101. doi: 10.1186/s12886-020-01369-1, 32169056 PMC7071674

[22] Ercan ZE Karalezli A Coban G. Pseudoduplication of the optic disc in moderate myopia. Saudi J Ophthalmol. (2016) 30:257–9. doi: 10.1016/j.sjopt.2016.11.003, 28003787 PMC5161814

[23] Lu H Wu Y Xiong J Zhou N Yamanari M Okamoto M . Whorl-like collagen fiber arrangement around emissary canals in the posterior sclera. Invest Ophthalmol Vis Sci. (2025) 66:35. doi: 10.1167/iovs.66.3.35, 40100203 PMC11927319

[24] Arlanzon-Lope P Campos MA Fernandez-Bueno I Coco-Martin RM. Does PLEX^®^ elite 9000 OCT identify and characterize most posterior pole lesions in highly myopic patients? J Clin Med. (2023) 12:1846. doi: 10.3390/jcm12051846 36902634 PMC10003842

[25] Padhi TR Samal B Kesarwani S Basu S Das T. Optic disc doubling. J Neuroophthalmol. (2012) 32:238–9. doi: 10.1097/WNO.0b013e3182464d9f, 22918263

[26] Gerth-Kahlert C Wildberger H. Optic disc doubling or pseudo-optic disc in colobomatous retinal abnormality? J Neuroophthalmol. (2013) 33:412. doi: 10.1097/WNO.0b013e31829bb026, 24145482

[27] Vedantham V. Double optic discs, optic disc coloboma, and pit: spectrum of hybrid disc anomalies in a single eye. Arch Ophthalmol. (2005) 123:1450–2. doi: 10.1001/archopht.123.10.1450, 16219745

[28] Andonegui J Aranguren M Garcia-Barberan H. Pseudodoubling of the optic disk. Retina. (2009) 29:715–6. doi: 10.1097/IAE.0b013e31819a981a, 19289988

[29] Freitas-da-Costa P Falcao M Carneiro A. Infrared reflectance pattern of macular pits in pathologic myopia. JAMA Ophthalmol. (2015) 133:e1580. doi: 10.1001/jamaophthalmol.2015.80, 26067697

[30] Hayreh SS. Posterior ciliary artery circulation in health and disease: the Weisenfeld lecture. Invest Ophthalmol Vis Sci. (2004) 45:748–9. doi: 10.1167/iovs.03-0469, 14985286

[31] Ohno-Matsui K Akiba M Moriyama M Shimada N Ishibashi T Tokoro T . Acquired optic nerve and peripapillary pits in pathologic myopia. Ophthalmology. (2012) 119:1685–92. doi: 10.1016/j.ophtha.2012.01.047, 22494632

[32] Xie S Fang Y Du R Yokoi T Takahashi H Uramoto K . Abruptly emerging vessels in eyes with myopic patchy chorioretinal atrophy. Retina. (2020) 40:1215–23. doi: 10.1097/IAE.0000000000002630, 31404035

[33] Chen H Liu X Zhou X Fu J Wang L. Advancements in myopic macular foveoschisis research. Ophthalmic Res. (2024) 67:424–34. doi: 10.1159/000540238, 38986459

